# Massive Scrotal Hematoma due to Ruptured Anastomotic Pseudoaneurysm in a Patient with Aortobifemoral Bypass Surgery: CTA Evaluation

**DOI:** 10.1155/2019/9013697

**Published:** 2019-11-24

**Authors:** Magdalini Smarda, Dimitrios Fagkrezos, Ilias Dodos, Anastasios Potouridis, Dimitrios Staramos, Charikleia Triantopoulou, Petros Maniatis

**Affiliations:** ^1^Department of Computed Tomography and Interventional Radiology, “Konstantopouleion” General Hospital of Nea Ionia, Athens, Greece; ^2^Department of Vascular Surgery, “Konstantopouleion” General Hospital of Nea Ionia, Athens, Greece; ^3^Department of Radiology, “Konstantopouleion” General Hospital of Nea Ionia, Athens, Greece

## Abstract

A 74-year-old male patient was presented with scrotal swelling and a pulsatile mass of the left femoro-inguinal region. His medical history included hypertension, coronary artery disease, respiratory failure, and an aortobifemoral bypass surgery performed 7 years ago. Ultrasound evaluation revealed a massive scrotal hematoma. Computed tomography angiography (CTA) was conducted, confirming the aortobifemoral graft existence and revealing bilateral anastomotic pseudoaneurysms with the left one being ruptured, resulting in extension of the hematoma to the left femoro-inguinal region and the scrotum. An emergency surgery was performed, where proximal control of the left limb of the synthetic graft as well as distal control of the iliac vessels were accomplished. After the control of the hemorrhage, an iliofemoral bypass with a Polytetrafluoroethylene (PTFE) 6 mm synthetic graft was placed. Unfortunately, the patient passed away during the first postoperative day due to myocardial infarction.

## 1. Introduction

Taking into account the large number of reconstructive vascular procedures being performed in latest clinical practice, there is an expected increase in the number of post-procedural complications, including anastomotic pseudoaneurysm formation. If those anastomotic pseudoaneurysms are left untreated, they may lead to peripheral embolism or rupture, conditions that are limb or life-threatening respectively and in great need for immediate intervention [1]. In this paper, we present a case of such a life-threatening rupture of anastomotic femoral artery pseudoaneurysm in a patient with an aortobifemoral bypass medical history. Written informed patient consent for possible future publication was obtained at the time of CTA performance.

## 2. Case Presentation

**Figure 1:**
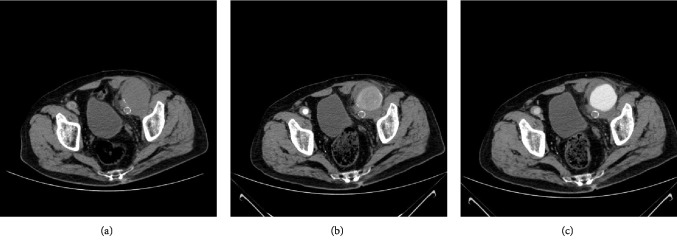
Same axial slice using a 64-detector row CT scanner (a) before and (b) & (c) after contrast media injection (arterial and portal phase respectively), revealing a ruptured left femoral pseudoaneurysm at the distal end of the left limb of the aortobifemoral graft.

**Figure 2:**
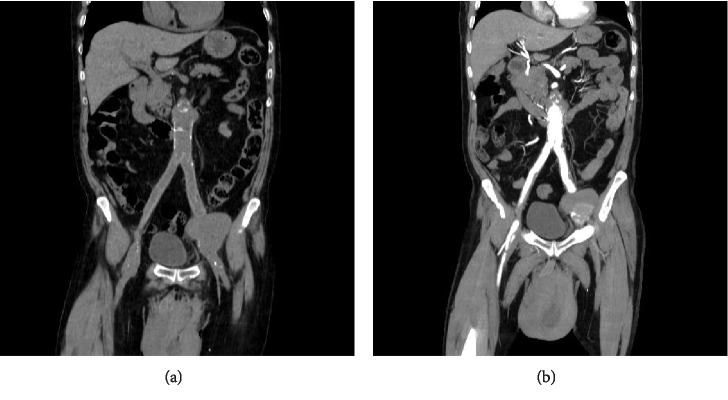
(a) & (b) Coronal MPR images before and after contrast media administration, showing both aortobifemoral graft presence and left anastomotic pseudoaneurysm rupture. Scrotal hematoma is also depicted.

**Figure 3:**
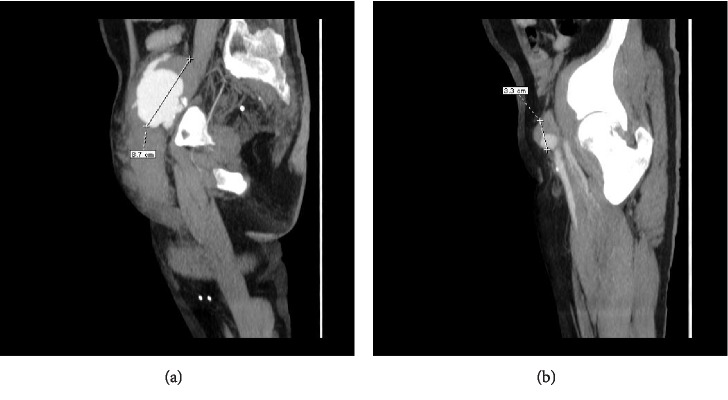
(a) & (b) Sagittal MPR images after contrast media administration (portal phase), revealing the maximum diameter of both anastomotic pseudoaneurysms, the left one (a) and the right one (b) respectively. Image (a) illustrates the femoro-inguinal hematoma as well.

**Figure 4:**
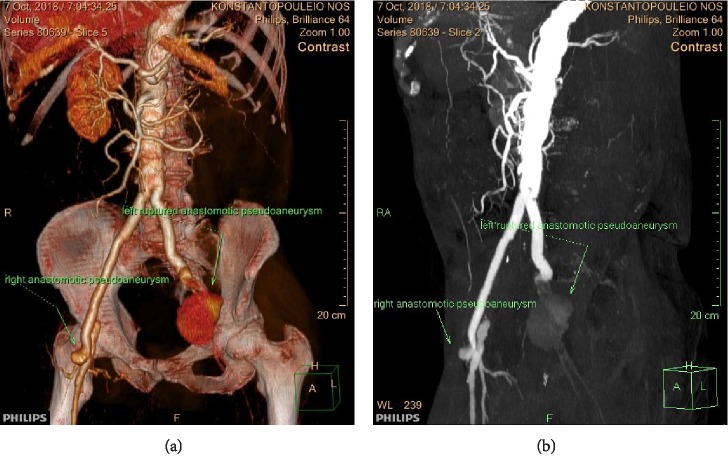
(a) VRT and (b) MIP reconstruction images respectively show both anastomotic pseudoaneurysms (the ruptured left one and the right one).

A 74-year-old male patient came to our hospital's ER with a progressively enlarging scrotal edema of non-traumatic nature, noticed during the last few hours. Clinical examination at the Urology Department revealed hemodynamic stability and a painful pulsatile mass of the left femoro- inguinal region without any existing signs of local or systemic infection, causing however extreme discomfort to the patient. His past medical history included hypertension, coronary artery disease and respiratory failure, whereas his surgical history of interest included an aortobifemoral bypass surgery using a synthetic graft due to severe peripheral arterial disease, conducted 7 years ago. Ultrasound evaluation of the scrotum revealed scrotal hematoma existence. A triple phase computed tomography angiography (CTA) examination of the abdomen and pelvis was then performed using our Department's 64-detector row CT scanner (Brilliance, Philips Healthcare, Cleveland, OH, USA) on an emergency basis, including unenhanced, arterial and portal phase post-IV contrast media administration. CT Angiography (CTA) protocol included supine patient positioning with hands above head and thin CT slices of 1.5 mm from the supraceliac aorta to proximal thighs. A total of 150 mL of non-ionic iodinated contrast material with iodine concentration of 370 mg/mL was used with a flow rate of 4 mL/sec, followed by saline flush. Both arterial and portal phase imaging were obtained using the fixed time-delay technique. Specifically, arterial phase CT angiographic images were obtained 25 sec after the onset of peripheral intravenous injection, whereas portal phase CT angiographic images were obtained 70 sec post contrast media injection. Maximum intensity projections (MIPs), oblique or curved multiplanar reformats and 3D volume-rendered (VR) images were also evaluated in addition to cross-sectional axial images. CTA showed the aortobifemoral graft and confirmed the clinical suspicion of a ruptured pseudoaneurysm at the distal end of the left limb of the graft (Figures 1 and 2). Specifically, it revealed a ruptured left anastomotic pseudoaneurysm measuring 8.7 cm in maximum diameter resulting in extension of the hematoma to the left femoro-inguinal region and the scrotum. The imaging examination also revealed another pseudoaneurysm at the distal end of the right limb of the aortobifemoral graft with a maximum diameter of 3.3 cm, but without apparent rupture (Figures 3 and 4). An incidental imaging finding was a left atrophic kidney, due to chronic renal artery thrombosis. The patient was immediately taken to the operating room, where an incision extending from the inguinal region to the lateral abdominal wall was performed. By using the retroperitoneal approach, the vascular team identified the retroperitoneal hematoma with the sac of the pseudoaneurysm. Proximal control of the left limb of the synthetic graft was accomplished and then a distal control of the iliac vessels. After the control of the hemorrhage an iliofemoral bypass was performed with a 6 mm PTFE graft (end-to-end from the left limb of the graft to the left common femoral artery). The patient was hemodynamically stable after the operation and was taken to the department for further hospitalisation. Unfortunately, the patient finally passed away during the first post-operative day due to myocardial infarction.

## 3. Discussion

In contrast to true aneurysms, which have all three layers of the arterial wall (intima, media and adventitia), pseudoaneurysms represent by definition an arterial wall defect that allows luminal arterial blood communication with the adjacent soft tissue [2–4]. They are usually a result of inflammation, trauma or iatrogenic causes such as surgical procedures. Among the iatrogenic pseudoaneurysms, the anastomotic ones appear at the site of the anastomosis of a vascular graft with the native vessel, after a reconstructive vascular procedure. They seem to result from suture line interruption between the graft material and the native vessel due to technical error, mechanical stress, native artery disease, or because of defects in either the prosthetic graft or the suture material itself [1, 4, 5]. Their pathogenesis has been associated with many local and systemic factors; systemic factors include high blood pressure, anticoagulation and atherosclerosis, whereas infection, trauma and mechanical pressure by the inguinal ligament are among the most important local ones [1]. In fact, in vascular prosthetic infections, coagulase-negative staphylococci appear as significant pathogens, whereas bacteria transported via lymphatic channels to the femoral canal may also have the same result [6, 7].

Although theoretically anastomotic pseudoaneurysms can develop at any anastomotic site, 80% of them appear at the femoro-inguinal region possibly because of acting forces from motion of the adjacent joint [8]. Among vessels, the femoral artery has the highest incidence of anastomotic pseudoaneurysm formation, ranging from 0.5 to 23.7% according to bibliography [1, 9]. Anastomotic pseudoaneurysms can present at any time after vascular reconstruction. Most frequently and especially when associated with local infection, they appear within the first 2 years. Otherwise, the etiological factor may be related with a long-standing infection mechanism [10, 11].

It is quite common for femoral pseudoaneurysms to measure up to 4 cm in diameter before being clinically identified and sometimes, they may exceed 15 cm before rupture [5]. Usually, asymptomatic anastomotic pseudoaneurysms or those with a maximum diameter of less than 2 cm exhibit no complications. Otherwise, they may result in serious conditions such as peripheral embolism or rupture, leading to limb or even life loss [12]. Indications for intervention include infection, rapid pseudoaneurysm expansion, skin necrosis, peripheral ischemia, pain and of course rupture [13].

According to the current guidelines, CTA represents the reference non invasive imaging modality for the diagnosis of anastomotic pseudoaneurysms and their associated complications. It can also reveal signs of impending pseudoaneurysm rupture (fissure existence or lack of thrombus homogeneity), and help at therapeutic approach planning [14, 15]. In comparison to Digital Subtraction Angiography (DSA), CTA is a faster, more accurate and less costly technique, more easily accessible to the patient. All the aforementioned advantages have rendered CTA the method of choice for the diagnosis of vascular pathology on an emergency basis [16]. Moving artifacts existence undoubtedly is a limiting factor, but inability of a patient to cooperate when being in a serious condition, is also a limiting factor for DSA. However, the fact that CTA is a well-accepted and less time-consuming diagnostic method than DSA, is its major advantage in cases of vascular emergency where early diagnosis is crucial for therapeutic procedure decision making, as in this case.

## 4. Conclusion

Anastomotic pseudoaneurysm rupture constitutes a vascular emergency condition in great need for immediate surgical treatment, since there is always the risk of death due to excessive hemorrhage. Whenever a patient with a pulsatile mass and a medical history of vascular bypass surgery comes to the ER, clinical suspicion of the above complication should exist and a CTA must always take place on an emergency basis, as it highlights the pathology and it also helps decide the appropriate therapeutic approach.
